# MEETING NEEDS: LINKING CLIENTS WITH A SELF-NEGLECT ALLEGATION TO HOME- AND COMMUNITY-BASED SERVICES

**DOI:** 10.1093/geroni/igae098.0142

**Published:** 2024-12-31

**Authors:** Samantha Tuft, Farida Ejaz, Courtney Reynolds

**Affiliations:** Benjamin Rose, Cleveland, Ohio, United States; Benjamin Rose Institute on Aging, Cleveland, Ohio, United States; Benjamin Rose, Cleveland, Ohio, United States

## Abstract

This paper describes results from a federal grant in which Oklahoma Adult Protective Services (APS) collaborated with the Oklahoma City Indian Clinic and gerontological researchers to enhance APS self-neglect practice. A total of 188 APS clients from 11 counties were interviewed by phone at baseline during the COVID pandemic. This paper focuses on a subset of 45 randomly selected clients who were offered extensive case management services for a period of four months beginning shortly after they were reported to APS. The clients were primarily white and female with a mean age of 62 years and average annual income between $12,000-$15,000. During the study, 24% of the clients were screened as experiencing self-neglect based on the Recognizing Abuse Tool (Ejaz, et al., 2017). More importantly, 76% of the 45 clients reported that they needed help in at least one area, resulting in a total of 175 identified needs. The top five most identified needs were home repairs/maintenance, in-home care, public benefits, transportation, and case management. We will describe whether client needs were met during the study along with barriers to service utilization. Findings will be explored in light of their implications for APS practice and policy, particularly the role time-limited, intensive case management can play in preventing further reports to APS.

